# Single image super-resolution with denoising diffusion GANS

**DOI:** 10.1038/s41598-024-52370-3

**Published:** 2024-02-21

**Authors:** Heng Xiao, Xin Wang, Jun Wang, Jing-Ye Cai, Jian-Hua Deng, Jing-Ke Yan, Yi-Dong Tang

**Affiliations:** 1 School of Computer Science and Information Security, Guilin University of Electronic Technology, Guilin, 541004 Guangxi China; 2 School of Information and Software Engineering, University of Electronic Science and Technology of China, Chengdu, 610000 Sichuan China; 3 School of Ocean Engineering, Guilin University of Electronic Technology, BeiHai, 536000 Guangxi China; 4 State Key Laboratory of Rail Transit Vehicle System, Southwest Jiaotong University, Chengdu, 610000 Sichuan China

**Keywords:** Computational neuroscience, Mathematics and computing, Computer science

## Abstract

Single image super-resolution (SISR) refers to the reconstruction from the corresponding low-resolution (LR) image input to a high-resolution (HR) image. However, since a single low-resolution image corresponds to multiple high-resolution images, this is an ill-posed problem. In recent years, generative model-based SISR methods have outperformed conventional SISR methods in performance. However, the SISR methods based on GAN, VAE, and Flow have the problems of unstable training, low sampling quality, and expensive computational cost. These models also struggle to achieve the trifecta of diverse, high-quality, and fast sampling. In particular, denoising diffusion probabilistic models have shown impressive variety and high quality of samples, but their expensive sampling cost prevents them from being well applied in the real world. In this paper, we investigate the fundamental reason for the slow sampling speed of the SISR method based on the diffusion model lies in the Gaussian assumption used in the previous diffusion model, which is only applicable for small step sizes. We propose a new Single Image Super-Resolution with Denoising Diffusion GANS (SRDDGAN) to achieve large-step denoising, sample diversity, and training stability. Our approach combines denoising diffusion models with GANs to generate images conditionally, using a multimodal conditional GAN to model each denoising step. SRDDGAN outperforms existing diffusion model-based methods regarding PSNR and perceptual quality metrics, while the added latent variable Z solution explores the diversity of likely HR spatial domain. Notably, the SRDDGAN model infers nearly 11 times faster than diffusion-based SR3, making it a more practical solution for real-world applications.

## Introduction

**Figure 1:**
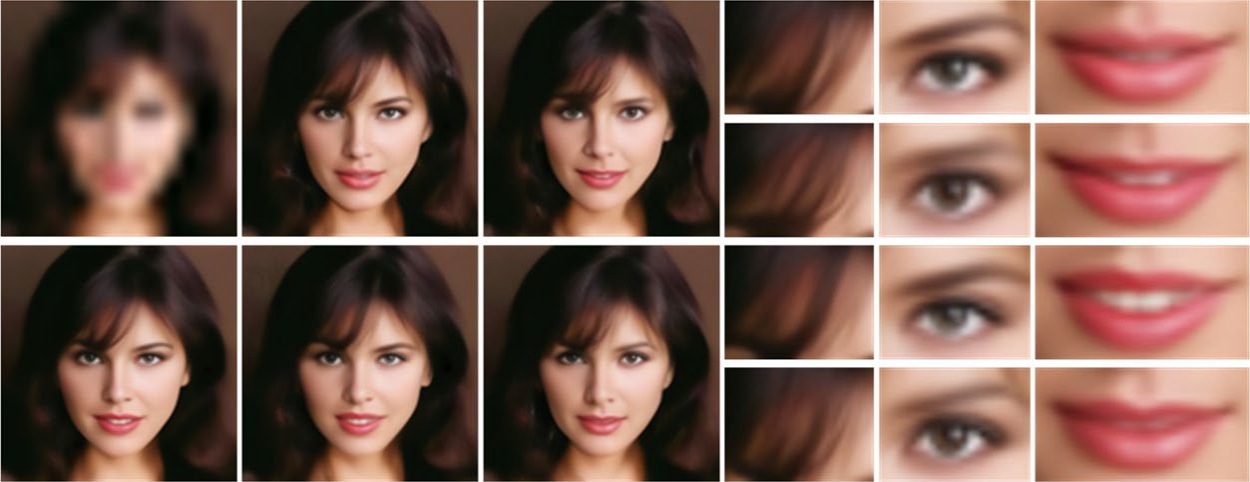
Random SR (8×) samples generated by SRDDGAN using latent variable Z. Our method generates diverse predicted SR images, including differences in facial attributes and hair (e.g., the second hair detail has a different texture than the fourth, and the third tooth being clearly visible while the fourth is not.), while maintaining consistency with the LR images.

Single Image Super-Resolution (SISR)^1^ refers to the process of reconstructing a high-resolution (HR) image from a low-resolution (LR) image, which is an essential technology in computer vision and image processing. It has a wide range of real-world applications, including remote sensing imaging^2^, video surveillance^3^, object detection^4^, and medical imaging^5,6^. As shown in Fig. 1, Super-Resolution is ill-posed^7,8^ and cannot be reversed by deterministic mapping because an infinite number of super-resolution images can be downsampled to the same low-resolution image. Instead, SISR can be described as learning a random mapping. When given a low-resolution image, this mapping reasonably randomly samples from its corresponding high-resolution image domain.

In order to establish the mapping between LR and HR, many generative model-based methods have emerged, which can be divided into five categories: Methods based on Autoregressive^9^, variational autoencoders (VAEs)^10^, Normalizing Flow^11^, Generative adversarial Networks (GANs)^12^, Based on the method of denoising diffusion probabilistic model (DDPM)^13,14^, however, these generative models all face three dilemmas: diversity of sampling, high quality of sampling, and fast sampling. Autoregressive-based methods, such as PixelCNN^15^, cannot be parallelized due to their pixel-by-pixel generation, resulting in slow sampling speed. The proposed method trains the model using the commonly used loss function (MSE). However, this may result in the sampled SR image being the average of multiple SR prediction results, reducing the diversity of the sampled images. VAE-based methods, such as CVAE^16^, where C is conditional, can use additional conditions to generate more diverse SR data, which can provide relatively fast sampling, but usually produce suboptimal sample quality. Normalizing Flows-based methods, such as SRFlow^17^, which adopts a reversible flow generation network structure, can learn the mapping relationship between the input LR image and the HR output image to realize image super-resolution. It uses negative log-likelihood loss for training to avoid training instability and mode collapse, but it is prone to large memory occupation and high sampling costs. GAN-based methods, such as SRGAN^18^, are commonly used networks for conditional image generation and super-resolution. These combine content and adversarial loss to reconstruct SR images with better perceptual quality. It can provide fast sampling but is prone to mode collapse, resulting in no diversity of generated SR samples and unstable training. Numerous researchers^19–21^ have suggested incorporating instance noise into the model input to address the instability in GAN model training. This helps to widen the solution space of the generator and discriminator, and improves the model’s resilience to overfitting.

Recently, denoising diffusion probabilistic models (commonly known as the diffusion model) have been recognized as powerful generative models due to their impressive performance in generating high-quality and diverse samples. The SISR method based on the diffusion model (e.g., SR3^22^, SRdiff^23^), which uses the Markov^24^ chain to transform the latent variable in Gaussian distribution into the data in complex distribution, solves the fundamental problem of the ill-posed SR and the quality of the sampled data is high. However, its main disadvantage is that the sampling speed is prolonged due to thousands of iteration steps. It makes them difficult to apply in the real world. Additionally, traditional denoising diffusion probabilistic models rely on unconditional or simple conditional inputs. In contrast, SISR tasks require a more thorough utilization of low-resolution images to restore high-frequency details fully within high-resolution images. In addition, it is well-known that when the degradation model presumed by an image super-resolution model does not match the actual image degradation^1,25,26^, it results in decreased model performance. Although studies have focused on specific degradation models (such as bicubic downsampling), they have yet to cover the diverse degradation modes in authentic images effectively.

In this paper, we investigate the problem of the slow sampling speed of SISR methods based on diffusion models. We note that diffusion models usually assume that the Gaussian distribution simulates the denoising distribution. However, the assumption that the denoising distribution is Gaussian leads to the inevitable small step size, which leads to many sampling steps and slow sampling speed. If we need to take a small number of sampling steps, this indicates that we need a denoising distribution that is de-parameterized with a non-Gaussian distribution. Following this heuristic, we propose to model the denoising distribution by multimodal distribution, which enables the denoising of giant steps.

Additionally, this paper introduces a more complex yet practical degradation model to address the challenge of inadequately covering the diverse degradation modes present in natural images. This model incorporates randomized permutations of blur, downsampling, and noise degradation to encompass a broader range of image degradation scenarios. Furthermore, to harness the valuable information within low-resolution images (LR) more effectively, we have employed a simple conditional generation approach and devised a specialized LR encoder module to constrain the high-resolution (HR) solution space. Lastly, style and content loss functions have been employed to restore certain high-frequency detail information.

In the SISR task, we introduced a novel conditional image generation approach called SRDDGAN. This method incorporates a multimodal distribution to model the denoising distribution and utilizes conditional GAN for modeling. Additionally, to adapt to diverse degradation modes, a more complex yet practical degradation model has been designed in this study. An LR Encoder module has been devised to utilize valuable information within low-resolution images (LR) efficiently. Moreover, instance noise injection has been implemented to foster stable GAN training and provide diversity. Furthermore, style and content loss functions have been utilized to restore high-frequency detail information. The new solution addresses current challenges and exhibits competitive sample quality and diversity in the SISR task compared to image super-resolution models based on diffusion models. Notably, our sampling process requires only four steps, approximately 11 times faster than diffusion models like SR3. Compared to traditional GANs, our proposed model significantly improves training stability and sample diversity while maintaining competitiveness in sample fidelity.

Our research has three main contributions:

1. We attribute the slow sampling of the diffusion model-based SISR method to the Gaussian distribution adopted in the denoising distribution and propose to employ a complex multimodal distribution to model the denoising distribution for fast sampling. Our approach produces images in just four steps, making it a competitive alternative to the most advanced models that require hundreds or thousands of sampling steps.
2. We propose SRDDGAN, which resolves the issue of unstable GAN training and sample diversity through instance noise injection, and its inverse process is parameterized by conditional GANs. SRDDGAN has introduced an intricate and pragmatic degradation model to tackle the various degradation modes found in genuine images.
3. We have created the LR Encoder module to limit the solution space of high-resolution images. This module extracts feature details from low-resolution images and transforms them into a latent space representation, used as input conditions for the model. Ultimately, we aim to improve the model’s fidelity and detail recovery by introducing style and content losses to restore and retain high-frequency details within the image.
4. The extensive experiments conducted on CelebA-HQ^27^, DIv2K^28^, and CIFAR10 datasets demonstrate the competitive performance of the proposed model in addressing the ill-posedness and fidelity of super-resolution images. SRDDGAN employs diffusion and reverse processes for flexible image manipulation, such as content fusion, and showcases its capability to handle complex degradation in real-world images.

## Background

This section is dedicated to the SISR task, initially presenting an overview of fundamental concepts associated with GAN and DDPM models. Subsequently, it introduces the theoretical foundation of our approach, which comprises four key components: first, reducing the sampling steps within the diffusion model; second, enhancing sample diversity by introducing instance noise, which is crucial for stabilizing GAN training. Additionally, it includes a complex and diverse degradation model. Finally, it ensured stable style and content consistency.

### GAN

Let us briefly review them to facilitate the understanding of Generative Adversarial Networks (GAN). GAN comprises two networks, a generator, and a discriminator, that learn through an adversarial process in which they play against each other. The ultimate goal of GAN is to use the max–min game^29^ between the two networks to simulate the actual data distribution (*p*(*x*)). The objective of the generative network in GAN is to convert random noise z into a distribution of actual data. In contrast, the discriminator network is trained to differentiate between actual samples ($x{\sim }p(x)$) and generated samples (*G*(*z*)). The two networks are constantly fighting and learning from each other. The ultimate goal is to make it unclear to the discriminator whether the result produced by the generator is accurate. The max–min game between the two networks can be expressed as follows.

1 $$\begin{aligned} {\min _{G} \max _{D} V(G,D)={{\mathbb {E}}}_{{\textbf{x}}\sim p({\textbf{x}})} [\log (D({\textbf{x}}))]}+ {{\mathbb E}}_{{\textbf{z}}\sim p({\textbf{z}})} [\log (1-D(G({\textbf{z}})))] \end{aligned}$$

However, it is worth noting that adversarial learning between G and D is typically kept constant despite potential issues such as instability during training and mode collapse that can arise when training GANs using the abovementioned formula. Various formula improvements have been proposed in practice^30^ to solve these problems.

### DDPM

To aid in the denoising diffusion probabilistic model, commonly known as the diffusion model, we will provide a brief overview of it. The diffusion model is a generative model that comprises two chains: a forward diffusion chain and an inverse diffusion chain.

**Forward diffusion chain:** The initial data distribution ${ x}_{0} \sim q(x_{0} )$ undergoes gradually adding Gaussian noise. As time t increases, it becomes an independent isotropic Gaussian distribution $x_{T}$. The mean value of the noise is determined by the data $x_{t}$ at the current time t and a fixed value $\beta _{t}$, while a fixed value $\beta _{t}$ determines the variance. This process is a Markov chain process^30^.

2 $$\begin{aligned}{} & {} q\left( {{\textbf{x}}_{t}}\mid {{\textbf{x}}_{t-1}} \right) =\mathscr{N}\left( {{\textbf{x}}_{t}};\sqrt{1-{{\beta }_{t}}}{{\textbf{x}}_{t-1}},{{\beta }_{t}}\textbf{I} \right) \end{aligned}$$

3 $$\begin{aligned}{} & {} q\left( {{\textbf{x}}_{1:T}}\mid {{\textbf{x}}_{0}} \right) =\prod \limits _{t=1}^{T}{q}\left( {{\textbf{x}}_{t}}\mid {{\textbf{x}}_{t-1}} \right) \end{aligned}$$

Specifically, at any time step t, $q(x_{t} )$ can be obtained directly from $x_{0}$ and $\beta _{t}$ without the need for iteration.

4 $$\begin{aligned} q\left( {{x}_{t}}\mid {{x}_{0}} \right) =\mathscr{N}\left( {{x}_{t}};\sqrt{{{{\bar{\alpha }}}_{t}}}{{x}_{0}},\left( 1-{{{\bar{\alpha }}}_{t}} \right) I \right) \qquad \text { where }{{\alpha }_{t}}:=1-{{\beta }_{t}},{{{\bar{\alpha }}}_{t}}:=\prod \limits _{s=1}^{t}{{{\alpha }_{s}}} \end{aligned}$$

**The reverse diffusion chain (denoised diffusion):** is constructed as

5 $$\begin{aligned}{} & {} {{p}_{\theta }}\left( {{\textbf{x}}_{0:T}} \right) =p\left( {{\textbf{x}}_{T}} \right) \prod \limits _{t=1}^{T}{{{p}_{\theta }}}\left( {{\textbf{x}}_{t-1}}\mid {{\textbf{x}}_{t}} \right) \end{aligned}$$

6 $$\begin{aligned}{} & {} {{p}_{\theta }}\left( {{\text {x}}_{t-1}}\mid {{\text {x}}_{t}} \right) =\mathscr{N}\left( {{\text {x}}_{t-1}};{{{\mu }}_{\theta }}\left( {{\text {x}}_{t}},t \right) ,\sigma _{t}^{2}\textbf{I} \right) \end{aligned}$$

The training process involves optimizing the typical variational lower bound on the negative logarithm of likelihood:

7 $$\begin{aligned} -\log {{p}_{\theta }}\left( {{\textbf{x}}_{0}} \right) \le -\log {{p}_{\theta }}\left( {{\textbf{x}}_{0}} \right) +{{D}_{\text {KL}}}\left( q\left( {{\textbf{x}}_{1:T}}\mid {{\textbf{x}}_{0}} \right) \Vert \right. \left. {{p}_{\theta }}\left( {{\textbf{x}}_{1:T}}\mid {{\textbf{x}}_{0}} \right) \right) ={{\mathbb {E}}_{q}}\left[ \log \frac{q\left( {{\textbf{x}}_{1:T}}\mid {{\textbf{x}}_{0}} \right) }{{{p}_{\theta }}\left( {{\textbf{x}}_{0:T}} \right) } \right] \end{aligned}$$

After taking the expectation on both sides of Eq. 7, we obtain the following:

8 $$\begin{aligned} {{L}_{{}}}={{\mathbb {E}}_{q}}\left[ \log \frac{q\left( {{\textbf{x}}_{1:T}}\mid {{\textbf{x}}_{0}} \right) }{{{p}_{\theta }}\left( {{\textbf{x}}_{0:T}} \right) } \right] \ge -{{\mathbb {E}}_{q}}\log {{p}_{\theta }}\left( {{\textbf{x}}_{0}} \right) \end{aligned}$$

The $L_{}$ can be further rewritten as:

9 $$\begin{aligned} {{L}_{{}}}={{\mathbb {E}}_{q}}[\underbrace{{{D}_{\text {KL}}}\left( q\left( {{\textbf{x}}_{T}}\mid {{\textbf{x}}_{0}} \right) \Vert {{p}_{\theta }}\left( {{\textbf{x}}_{T}} \right) \right) }_{{{L}_{T}}}+ \text { }\sum \limits _{t=2}^{T}{\underbrace{{{D}_{\text {KL}}}\left( q\left( {{\textbf{x}}_{t-1}}\mid {{\textbf{x}}_{t}},{{\textbf{x}}_{0}} \right) \Vert {{p}_{\theta }}\left( {{\textbf{x}}_{t-1}}\mid {{\textbf{x}}_{t}} \right) \right) }_{{{L}_{t-1}}}}- \text { }\underbrace{\log {{p}_{\theta }}\left( {{\textbf{x}}_{0}}\mid {{\textbf{x}}_{1}} \right) }_{{{L}_{0}}}] \end{aligned}$$

In the equation above, there are two parts: $L_{0}$ and $L_{T}$. Since the original paper^14^ chose a fixed variance, $L_{T}$ is a constant. On the other hand, $L_{0}$ is processed using the method described in the original DDPM paper, which involves discretizing the continuous Gaussian distribution. The formula for this conversion can be found in^13^, which also yields a constant value for $L_{0}$. Therefore, we can further process the *L* as follows:

10 $$\begin{aligned} L=\sum \limits _{t=2}^{T}{{{D}_{\text {KL}}}}\left( q\left( {{\textbf{x}}_{t-1}}\mid {{\textbf{x}}_{t}},{{\textbf{x}}_{0}} \right) \Vert {{p}_{\theta }}\left( {{\textbf{x}}_{t-1}}\mid {{\textbf{x}}_{t}} \right) \right) +C \end{aligned}$$

Ultimately, our training objective translates to minimizing Eq. 10, where C is a constant.

Diffusion models commonly adopt the Gaussian distribution as a denoising distribution, requiring hundreds to thousands of steps. However, our paper specifically concentrates on a diffusion model that involves a smaller number of steps.

### Large step denoising with multimodal distribution

Sampling speed is one of the main obstacles currently hindering the practical application of diffusion models^13,14,22^. The diffusion model typically assumes that the Gaussian distribution approximates the true denoising distribution $q(x_{t-1} |x_{t} )$. As per the Bayes formula^31^, the denoising distribution $q(x_{t-1} |x_{t} )$ can be expressed as $q(x_{t-1} |x_{t} ){\propto } q(x_{t} |x_{t-1} )q(x_{t-1} )$, where $q(x_{t} |x_{t-1} )$ represents the forward diffusion chain and $q(x_{t-1} )$ represents the edge probability. Assuming that the denoising distribution follows a Gaussian distribution, it is valid in specific scenarios. When $\beta _{t}$ is sufficiently tiny at each step, $q(x_{t} |x_{t-1} )$ dominates the Bayesian transformation equation, resulting in the reverse diffusion chain having the same functional form as the forward diffusion chain^32^. As a result, if the forward diffusion is Gaussian, the reverse diffusion will also be Gaussian. However, diffusion models often necessitate hundreds or thousands of steps with small $\beta _{t}$ to meet this condition, leading to slow sampling.

**Figure 2:**
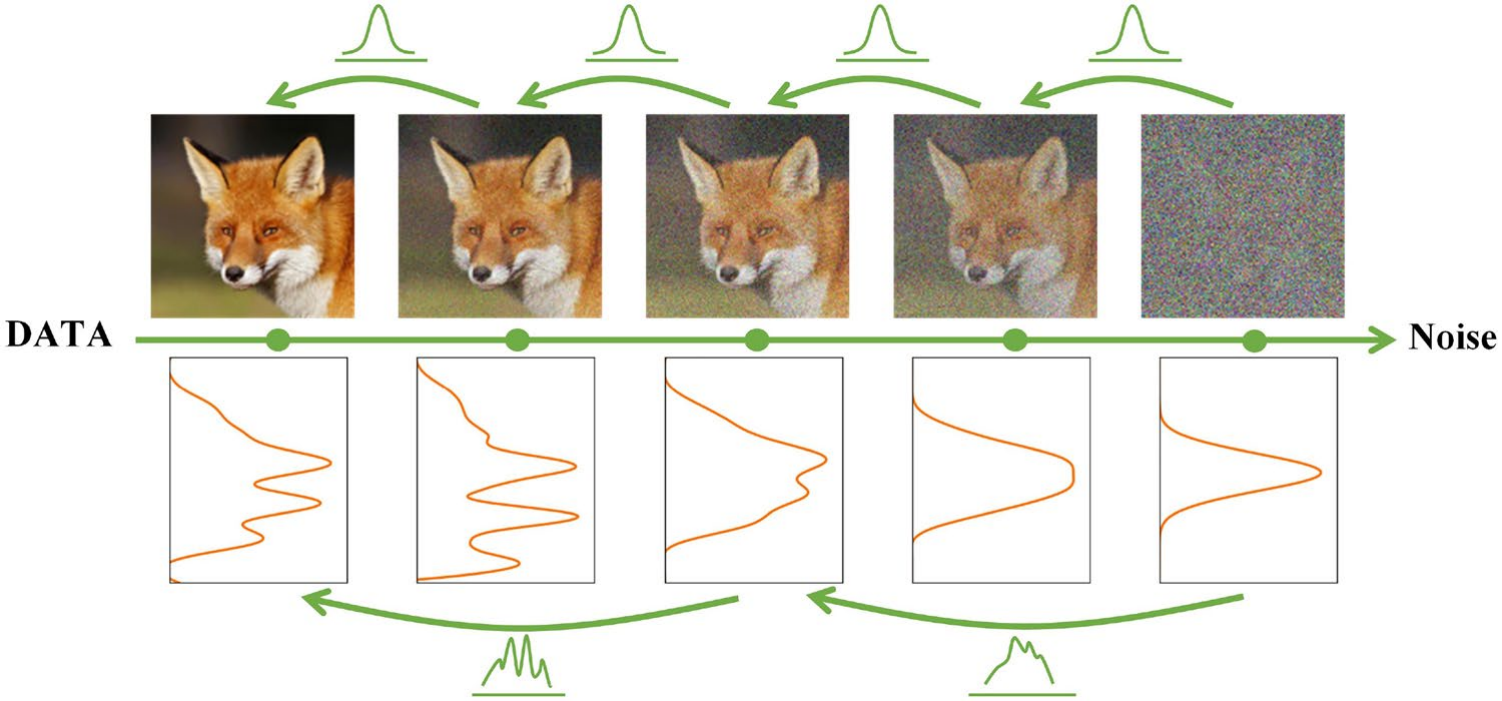
Middle:We systematically introduce Gaussian noise to the initial data distribution during the forward diffusion process, gradually transforming it into an independent isotropic Gaussian distribution. Top: When denoising, the model’s step size is set to a very small value if a Gaussian distribution is assumed to be used for the task. Bottom: However, increasing the step size leads to a more complex and multimodal denoising distribution, which can significantly accelerate the sampling speed.

When $\beta _{t}$ is sufficiently large, the assumption that the denoised distribution follows a Gaussian distribution is no longer valid. As $\beta _{t}$ increases, the step size of the denoising distribution will also increase, leading to a reduction in the required steps and a faster sampling speed. Therefore, a more complex multimodal distribution is necessary to model the denoising distribution instead of using a Gaussian distribution. From Fig. 2, it is evident that as the step size of the denoising distribution increases, the denoising distribution becomes progressively more complex and multimodal.

### Conditional GAN

SISR is commonly described as learning a random mapping between high-resolution (HR) and low-resolution (LR) images. However, the original diffusion model used in building the denoising distribution $p_{\theta } (x_{t-1} |x_{t} )$ predicts ${ x}_{0}$ from $x_{t}$ deterministically through iterative processes, which deviates from the desired random mapping. Our approach, on the other hand, generates the denoising distribution by passing through the generator with a latent random variable z. As a result, our denoising distribution $p_{\theta } (x_{t-1} |x_{t} )$ is more complex and multimodal than the original one.

To fit the noise model with a complex multimodal distribution, we increase the step size of the step and reduce the number of samples. Since conditional GANs^33^ can model complex distributions, we use them to fit the denoising distribution.

Injecting instance noise into the generator has been identified as an integral approach to enhancing the stability of GAN training and reducing overfitting induced by the discriminator focusing on pure data. It is apparent from the available literature^19,20^ on GAN that incorporating noise into the generator enhances the stability of GAN training. Thus, the incorporation of noise has become a prevalent technique for achieving both the stability of GAN training and a diverse range of generated samples.

### Diverse forms of degradation

**Figure 3:**
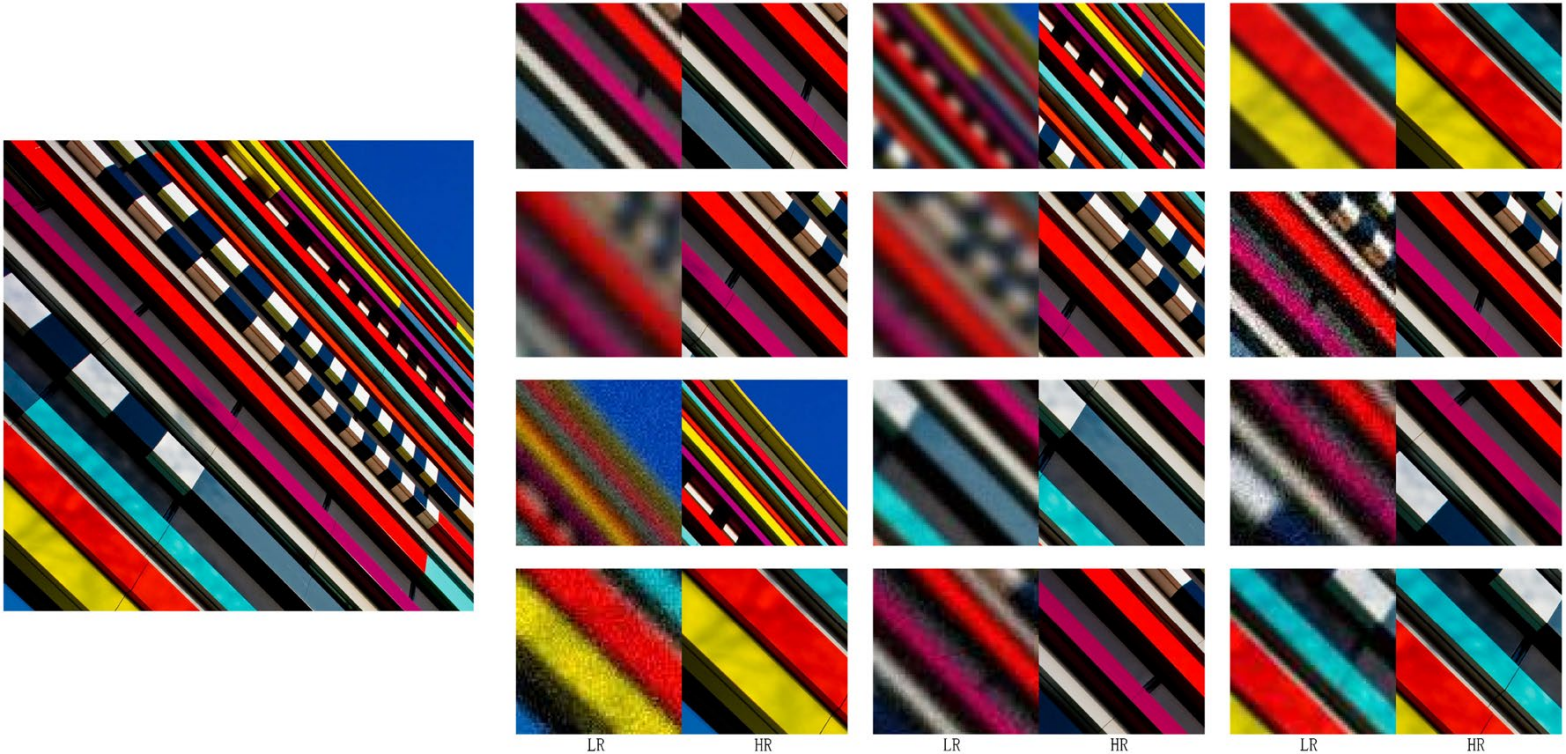
To address the diverse degradation modes present in the authentic image.

Following the relevant literature^1,25,26^, this study employs various degradation methods to process obtained low-resolution (LR) images, aiming to address the diverse and complex degradation scenarios encountered in the real world. Our approach encompasses a range of processing strategies, such as blurring, downsampling, and noise addition. Blurring degradation includes two types: isotropic Gaussian blur and anisotropic Gaussian blur. Downsampling degradation employs methods like nearest-neighbor, bilinear, and bicubic interpolation to simulate the effect of reducing image size. Noise degradation replicates various image noise types, including Gaussian noise, JPEG compression, and camera sensor noise. Combining these methods generates the final LR image. This diversified degradation approach enhances the model’s adaptability to various imperfect inputs, resulting in higher-quality super-resolution images. The results of our diversified degradation are shown in Fig. 3.

11 $$\begin{aligned} {{X}_{LR}}=({I}\times k){{\downarrow }_{s}}+n \end{aligned}$$

Where ${X}_{LR}$ denotes low-resolution, while *I* denotes the image undergoing processing. The variable *k* symbolizes the blur kernel that simulates potential blurriness during image capture. The symbol $\times$ signifies the convolution operation. The $\downarrow$ notation indicates downsampling, where *s* represents the downsampling factor. Lastly, *n* represents the noise added to the image.

### Consistency of style and content

In the SISR task, a single low-resolution image might correspond to multiple high-resolution images, presenting an ill-posed problem. Initially, using L1 or L2 loss during training frequently led to blurred predictions despite yielding higher PSNR metrics. This approach leans toward average losses, inadequately addressing uncertainty in super-resolution problems, resulting in a notable decrease in high-frequency details. Recently, leveraging VGG-19’s^26,34,35^ style and content losses has demonstrated the ability to generate more explicit images and improve visual quality, notably assisting in restoring high-frequency details.

**Content loss**: Content loss^1,25,26^ is introduced into the SISR task to evaluate the perceptual quality of images. Specifically, we employ a pre-trained classification network to measure the semantic differences between images. This network is denoted as $\phi$, and the high-level representations extracted at layer *l*-th are represented as ${{\phi }^{(l)}}(I)$. The content loss is defined as the Euclidean distance between the high-level representations of the two images, as shown below:

12 $$\begin{aligned} {{L}_{\text {content }}}(\hat{I},I;\phi ,l)=\frac{1}{{{h}_{l}}{{w}_{l}}{{c}_{l}}}\sqrt{\sum \limits _{i,j,k}{{{\left( \phi _{i,j,k}^{(l)}(\hat{I})-\phi _{i,j,k}^{(l)}(I) \right) }^{2}}}} \end{aligned}$$

Where ${{h}_{l}}$, ${{w}_{l}}$, and ${{c}_{l}}$ represent the height, width, and number of channels of the representations on layer *l*, respectively.

**Style loss**: As reconstructed images should exhibit a similar style to the target image (e.g., color, texture, contrast), inspiration from style representations is drawn. Style loss (texture loss)^1,25,26^ is introduced into the SISR task. The style of an image is regarded as the correlation between different feature channels. It is defined as the Gram matrix $G_{ij}^{(l)}\in {{R}^{ci\times cj}}$, where $G_{ij}^{(l)}$ denotes the inner product between vectorized feature maps *i* and *j* at layer *l*. The formula is represented as follows:

13 $$\begin{aligned} G_{ij}^{(l)}(I)={\text {vec}}\left( \phi _{i}^{(l)}(I) \right) \cdot {\text {vec}}\left( \phi _{j}^{(l)}(I) \right) \end{aligned}$$

Where *vec*(.) denotes the vectorization operation, and $\phi _{i}^{(l)}(I)$ represents the *i*-th channel in the *l*-th feature map of the image (I). Therefore, the style loss is expressed as:

14 $$\begin{aligned} {{L}_{style}}(\hat{I},I;\phi ,l)=\frac{1}{c_{l}^{2}}\sqrt{\sum \limits _{i,j}{{{\left( G_{i,j}^{(l)}(\hat{I})-G_{i,j}^{(l)}(I) \right) }^{2}}}} \end{aligned}$$

## Method

This section presents our proposed Single Image Super-Resolution (SISR) task model, the Conditional Denoising Diffusion GANS Model (SRDDGAN). The section begins by providing a brief introduction to the fundamental concept of the model. Subsequently, a detailed description of the forward diffusion process is presented. Furthermore, this section provides comprehensive insights into our model’s training and optimization process, culminating with a detailed explanation of how to extrapolate our denoising model.

### Conditional Denoising Diffusion GANS Model

For the SISR task, a high-resolution (HR) image dataset and its corresponding low-resolution (LR) counterpart are combined to create a paired dataset $D=\{ x_{i},y_{i} \} _{i=1}^{N}$, representing samples obtained from a distribution *p*(*y*|*x*) with unknown properties. This dataset has an ill-posed mapping between LR and HR images, meaning that a single low-resolution source image x may correspond to multiple high-resolution target y. Our objective is to acquire the capability to generate high-resolution images that closely match distribution *p*(*y*|*x*), given a low-resolution image as input.

A denoising model based on a complex multimodal distribution was utilized to effectively deal with the instability issues associated with GAN training and learn the ill-posed mapping between LR and HR images. The proposed method involves the denoising model (DDPM) and generative adversarial network (GAN) for conditional image generation aimed at resolving these challenges.

**Figure 4:**
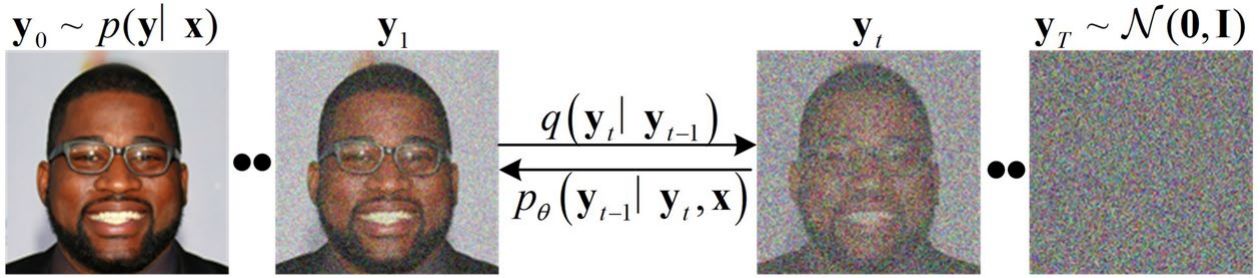
The forward diffusion process involves gradually adding Gaussian noise to the original image, progressing from left to right until it becomes a fully Gaussian noise distribution. In contrast, the reverse diffusion process proceeds from right to left, utilizing the source image x as the condition for iterative denoising.

The Conditional Denoising Diffusion GANs model can generate the target image $y_{0}$ in a relatively small number of iteration steps T. Starting from purely Gaussian noise, the model leverages conditional transfer learning to generate samples from the distribution $p_{\theta } (y_{t-1} |y_{t},x,z)$, where x denotes the source image and z represents potential random variables. By iterating through detailed images in sequence $(y_{t-1} ,y_{t-2} ,...,y_{0} )$, the model eventually converges to the point where $y_{0} \infty p(y|x)$. Refer to Fig. 4 for a visual representation. Note that the source image x is not displayed in this illustration.

Our model assumes a small value for T and defines the distribution of intermediate images in the inference chain using a forward diffusion process. At each diffusion step, a large $\beta {t}$ is required (See Appendix B for specific B settings). This process involves the gradual addition of Gaussian noise to the original data through a fixed forward diffusion chain, denoted as $q(y_{t} |y_{t-1} )$ (Fig. 4). Our model aims to recover the original data distribution iteratively from noise through a reverse diffusion chain, conditioned on both the source image x and the noisy image. We train a neural denoising model *G* to learn the reverse diffusion chain to achieve this. The denoising model denoted as *G* is presented with inputs, namely a source image, a noisy image, and a latent variable Z, which predicts the output image($y_0$).

The following sections overview the forward diffusion process and describe how our denoising model *G* is trained and inferred.

### Forward diffusion process

Following the literature^13,14,21,31^, we establish our forward diffusion chain using a method similar to the diffusion process described in Eqs. 2 and 3 Specifically, we can employ Eq. 4 for the forward diffusion.

15 $$\begin{aligned} q\left( {{y}_{t}}\mid {{y}_{0}} \right) =\mathscr{N}\left( {{y}_{t}};\sqrt{{{{\bar{\alpha }}}_{t}}}{{y}_{0}},\left( 1-{{{\bar{\alpha }}}_{t}} \right) I \right) \qquad \text { where }{{\alpha }_{t}}:=1-{{\beta }_{t}},{{{\bar{\alpha }}}_{t}}:=\prod \limits _{s=1}^{t}{{{\alpha }_{s}}} \end{aligned}$$

It is worth noting that our approach differs from previous diffusion models, which typically require thousands of steps. In our method, we assume that T is small, which means that $\beta _{t}$ at each diffusion step is large enough.

One can obtain the posterior distribution from Eq. 16 given the $y_{0}$ and $y_{t}$, as shown below:

16 $$\begin{aligned} q\left( {{\textbf{y}}_{t-1}}\mid {{\textbf{y}}_{0}},{{\textbf{y}}_{t}} \right) =\mathscr{N}\left( {{\textbf{y}}_{t-1}}\mid \varvec{\mu }_{\textbf{t}},{{\sigma }^{2}}\textbf{I} \right) \end{aligned}$$

where the mean and variance in $q(y_{t-1} |y_{t},y_{0} )$ are obtained from Eqs. 17 and 18.

17 $$\begin{aligned} {{\mu }_{t}}= & {} \frac{\sqrt{{{\alpha }_{t}}}\left( 1-{{{\bar{\alpha }}}_{t-1}} \right) }{1-{{{\bar{\alpha }}}_{t}}}{{\textbf{y}}_{t}}+\frac{\sqrt{{{{\bar{\alpha }}}_{t-1}}}{{\beta }_{t}}}{1-{{{\bar{\alpha }}}_{t}}}{{\textbf{y}}_{0}} \end{aligned}$$

18 $$\begin{aligned} {{\sigma }^{2}}= & {} \frac{1-{{{\bar{\alpha }}}_{t-1}}}{1-{{{\bar{\alpha }}}_{t}}}\cdot {{\beta }_{t}} \end{aligned}$$

The posterior distribution plays a dual role in parameterizing the reverse diffusion chain and formulating a variational lower bound on the log-likelihood of the chain. Moving forward, we will explore using generative adversarial networks to parameterize this denoising model.

### Optimizing the Denoising Diffusion GANS Model

To facilitate the inverse diffusion process, we adopt the approach proposed in previous work^22,23,31^, where a neural network *G* is trained using supplementary information from the input image *x*. Specifically, the network takes as inputs a noisy target image $y_t$ and a source image *x*, and its objective is to reconstruct a clean version of the target image by removing the noise, as described in Eq. 19.

19 $$\begin{aligned} {{y}_{t}}=\sqrt{{{{\bar{\alpha }}}_{t}}}{{\textbf{y}}_{0}}+\sqrt{1-{{{\bar{\alpha }}}_{t}}}\epsilon ,\quad \epsilon \sim \mathscr{N}(\textbf{0},\textbf{I}) \end{aligned}$$

To be precise, our denoising model G requires the input of not only the source image *x* and the noisy target image $y_{t}$, but also the latent variable Z ($Z\sim N(0, I)$)) and t ($t \sim U(1,T)$). During training, our goal is to minimize the adversarial loss, as demonstrated in Eq. 10, which is comparable to the one presented in the previous section. To express our loss in a different form, we have rephrased it in Eq. 20 by applying the equivalent transformation of L as detailed in Appendix A of DDPM^14^.

20 $$\begin{aligned} L=\sum \limits _{t\ge 1}{{{\text {E}}_{q\left( {{\text {y}}_{t}} \right) }}}\left[ {{D}_{\text {adv }}}\left( q\left( {{\textbf{y}}_{t-1}}\mid {{\text {y}}_{t}} \right) \Vert {{p}_{\theta }}\left( {{\text {y}}_{t-1}}\mid {{\text {y}}_{t}} \right) \right) \right] \end{aligned}$$

The adversarial loss $D_{adv}$ in GAN can be formulated using different types of divergence measures, such as KL divergence, Jensen-Shannon divergence, and others^36^. However, for this particular case, the f-divergence has been chosen.

In adversarial training, the approach is akin to the training process of most GANs. The traditional method of training the discriminator in GANs involves using the input $y_{0}$, which exposes it to a surplus of clean data and can lead to overfitting. However, in our model, we have designed the discriminator to receive noisy target images $y_{t}$ and $y_{t-1}$ as input. This critical difference in the training process makes our model more stable compared to the original GAN.

Specifically, the discriminator $D(y_{t-1}, y_{t},t)$ takes two noisy target images $y_{t-1}$ and $y_{t}$ as inputs and outputs the confidence score that $y_{t-1}$ is a denoised version of $y_{t}$. Adversarial training as in Eq. 21

21 $$\begin{aligned} {{L}_{adv}} = \sum _{t=1} \mathbb {E}_{q(y_t)}\left[ \mathbb {E}_{q(y_{t-1}|y_t)}\left[ -\log \left( D(y_{t-1},y_t,t) \right) \right] \right] +\mathbb {E}_{p_{\theta }(y_{t-1}|y_t)}\left[ -\log \left( 1 - D(y_{t-1}',y_t,t) \right) \right] \end{aligned}$$

The objective of the discriminator is to maximize its confidence in identifying a sample from the true distribution ${ q}(y_{t-1} |y_{t} )$ while minimizing its confidence in identifying a fake sample from $p_{\theta } (y_{t-1} |y_{t} )$. Conversely, the generator aims to increase the likelihood that the fake samples it produces are classified as genuine by the discriminator. Please note that the formula above requires an unknown distribution, ${q}\left( {{y}_{t-1}}\left| {{y}_{t}} \right. \right)$, in order to obtain samples. However, we can use the identity ${q}\left( {{y}_{t-1}}\left| {{y}_{t}} \right. \right) :=\int {q\left( {{y}_{0}} \right) q\left( {{y}_{t}},{{y}_{t-1}}\left| {{y}_{0}} \right. \right) }d{{y}_{0}}=\int {q\left( {{y}_{0}} \right) q\left( {{y}_{t-1}}\left| {{y}_{0}} \right. \right) }q\left( {{y}_{t-1}}\left| {{y}_{t}} \right. \right) d{{y}_{0}}$ in order to express it in terms of what we already know. Moreover, concerning the denoising model $p_{\theta } (y_{t-1} | y_{t})$ in diffusion models, it has been proposed by^14^ that the denoising model can be parameterized as $p_{\theta } (y_{t-1} | y_{t}):=q(y_{t-1} |y_{t},y_{0})$.

Our approach differs from previous methods^13,21,22^ in that we return the generator output to the forecast $y_{0}$ instead of the prediction noise. Although the noise and $y_{0}$ values can be converted into each other based on $\bar{\alpha }_{t}$ and $y_{t}$ (Eq. 19), we directly predict $y_{0}$ using the generator, which simplifies the model’s transformation step and accelerates the inference process. This is what sets our diffusion model algorithm apart from others.

Finally, we employed VGG-19’s(relu1.2, relu2.2, relu3.3, and relu4.1) style and content losses to recover high-frequency details in super-resolution image reconstruction. Following relevant literature^26,35,37^, our utilization of VGG-19 content loss involves extracting content features from input and target images using a neural network and computing the distance between these features. Meanwhile, the style loss involves extracting style features from input and target images using a neural network and computing the distance between these features. The model is trained by combining these loss functions. The overall loss function of the model is depicted in Eq. 22.

22 $$\begin{aligned} {{L}_{total}}=\alpha {{L}_{adv}}+\beta {{L}_{content}}+\eta {{L}_{style}} \end{aligned}$$

**Figure 5:**
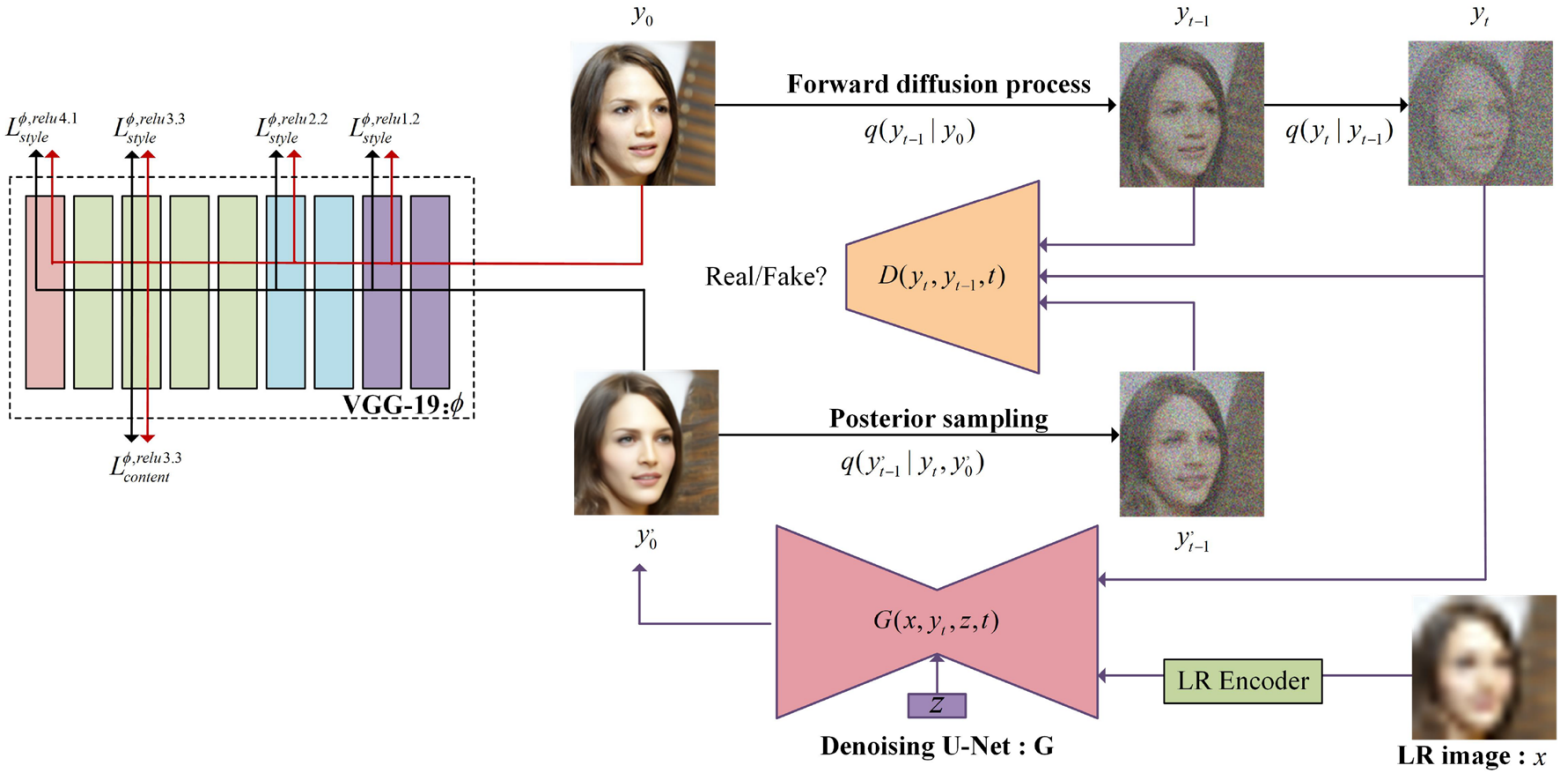
The training process of SRDDGAN.

Where ${L}_{adv}$ denotes the foundational loss of the SRDDGAN model, while ${L}_{content}$ and ${L}_{style}$ refer to the reduction of style and content losses in super-resolved images based on a pre-trained VGG-19 model. The weights $\alpha$, $\beta$, and $\eta$ signify the importance of each loss function. The training process can be illustrated through Fig. 5.

### Inference

To perform inference in our model, we initiate the process in the reverse direction of the forward diffusion process, starting from pure Gaussian noise $y_{T}$.

23 $$\begin{aligned}{} & {} {{p}_{\theta }}\left( {{\textbf{y}}_{0:T}}\mid \textbf{x} \right) =p\left( {{\textbf{y}}_{T}} \right) \prod \limits _{t=1}^{T}{{{p}_{\theta }}}\left( {{\textbf{y}}_{t-1}}\mid {{\textbf{y}}_{t}},\textbf{x} \right) \end{aligned}$$

24 $$\begin{aligned}{} & {} p\left( {{\textbf{y}}_{T}} \right) =\mathscr{N}\left( {{\textbf{y}}_{T}}; \textbf{0},\textbf{I} \right) \end{aligned}$$

25 $$\begin{aligned}{} & {} {{p}_{\theta }}\left( {{\textbf{y}}_{t-1}}\mid {{\textbf{y}}_{t}},\textbf{x} \right) =\mathscr{N}\left( {{\textbf{y}}_{t-1}}\mid {{\mu }_{\theta }}\left( \textbf{x},{{\textbf{y}}_{t}},\textbf{z},\textbf{t} \right) ,\sigma _{t}^{2}\textbf{I} \right) \end{aligned}$$

Our inference procedure is based on the complex multimodal distribution $p_{\theta } (y_{t-1} |y_{t},x)$ learned by the model. Referring to the theory in the previous section, when the forward diffusion $\beta _{t}$ is set to the possible maximum value, the optimal denoising distribution $p_{\theta } (y_{t-1} |y_{t},x)$ approximates a distribution of multiple peaks. Therefore, our inference process incorporates the conditions of a multimodal distribution, which can reasonably fit the reverse diffusion process. As per Eq. 15, A should be as small as possible when $\beta _{t}$ is set large enough so that $y_{t}$ approximates a Gaussian distribution^13^, and Eq. 24 can be obtained. Sampling can start from pure Gaussians.

To predict $y_{t-1}$ directly during the denoising stage, we employ a technique akin to that used in^13,14^. First, the model *G* is trained for denoising to estimate the value of $y_{0}^{\prime }$ after we feed the source image *x*, the noisy image $y_{t}$, the temporal variable *t*, and *z* into it. Then, we use the estimated value of $y_{0}^{\prime }$ to derive the posterior distribution $q(y_{t-1} |y_{t},y_{0})$ using equations (Eqs. 17 and 18). Finally, we use this posterior distribution to parameterize the mean and variance of the parametric distribution $p_{\theta } (y_{t-1} | y_{t}, x)$ (Eqs. 26 and 27).

26 $$\begin{aligned}{} & {} {{\mu }}_{\theta }=\frac{\sqrt{{{\alpha }_{t}}}\left( 1-{{{\bar{\alpha }}}_{t-1}} \right) }{1-{{{\bar{\alpha }}}_{t}}}{y}_{t}+\frac{\sqrt{{{{\bar{\alpha }}}_{t-1}}}{{\beta }_{t}}}{1-{{{\bar{\alpha }}}_{t}}}{{y}_{0}^{\prime }} \end{aligned}$$

27 $$\begin{aligned}{} & {} {{\sigma }^{2}}=\frac{1-{{{\bar{\alpha }}}_{t-1}}}{1-{{{\bar{\alpha }}}_{t}}}\cdot {{\beta }_{t}} \end{aligned}$$

Notably, the variance used here employs the default values provided by the forward diffusion variance^14^.

Similar to the approach in the paper^13,14^, we employ a reparameterization trick^10^ to refine the model iteratively. The specific form of this technique is as follows:

28 $$\begin{aligned} {{\textbf{y}}_{t-1}}={{\mu }}_{\theta }+\sigma {{\varepsilon }_{t}} \qquad \text {where } {{\varepsilon }_{t}}\sim \mathscr{N}(\textbf{0},\textbf{I}) \end{aligned}$$

This step is akin to Langevin dynamics^13^, where we iteratively refine the inference by following Eq. 28 and ultimately obtain the denoised image.

**Algorithm 1:**
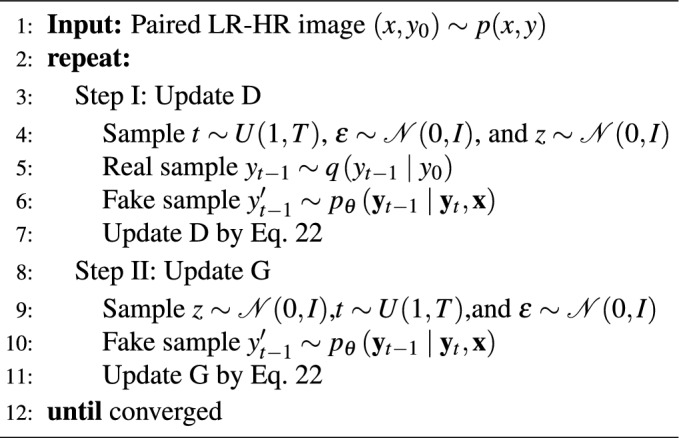
Training a denoising model G

**Algorithm 2:**
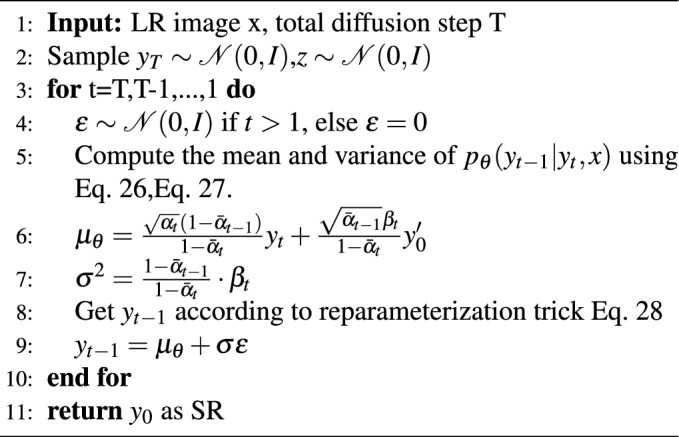
Inference in T iterative refinement steps

### Informed Consent

The images included in our study are sourced from a publicly available dataset that contains facial data. These images were collected and made publicly accessible by the dataset provider, who ensured compliance with the relevant usage rules and guidelines. As the authors of this study, we have strictly adhered to these rules and guidelines while using the dataset for our experiments.

## The Structure Of The SRDDGAN Model

This section will outline the model structure of SRDDGAN, which consists of both generators and discriminators and the number of denoising steps utilized.

Our model’s generator architecture resembles the U-net architecture utilized in NCSN++^36^, which comprises several residual blocks and attention blocks. The sinusoidal position function regulates the time step, as per DDPM. We employ the residual blocks from BiGAN^29^ instead of the original DDPM’s residual blocks, increasing their number. Following StyleGAN^37^, we also incorporate latent variable z conditions in the U-net architecture, which sets our generator apart from previous diffusion model networks. Specific settings, such as the Swish activation function, can be found in the original paper. To confine the solution space of high-resolution images, we developed the LR Encoder module capable of extracting feature details from the low-resolution image and transforming them into a latent space representation. In the subsequent section, Table 1 presents examples of hyperparameter designs for generator networks, such as the number of blocks and initial channel number. (see Appendix A for details).

Crucially, this paper introduces the utilization of an LR Encoder that processes LR information and integrates it into each reverse diffusion step to steer the generation toward the corresponding HR space. We opted for an RRDB^38^ architecture inspired by SRFlow^17^, renowned for its residual-in-residual design and numerous dense skip connections. However, we have removed the final convolutional layer from the RRDB architecture as we aim not to obtain SR outcomes but rather to concentrate on the concealed LR image particulars. Additionally, we have removed the BN layers due to findings in pertinent literature^1,25,38^ indicating their potential to introduce unwanted artifacts and constrain the model’s capacity for generalization.

We take a comparable approach^1^ and create our discriminator with a convolutional neural network using ResNet blocks, which are designed similarly to generators. The discriminator aims to discriminate between true and false $y_{t-1}$, using $y_{t}$ and t as contextual conditions. We incorporate time adjustment by utilizing sinusoidal position embedding, also employed in the generator. To adjust $y_{t}$ for input to the discriminator, we arrange $y_{t}$ and $y_{t-1}$ in series. (see Appendix A for details).

The diffusion model presented in previous research^13,14^ often required hundreds or thousands of diffusion steps during inference, resulting in slow image synthesis. Multiple improvements have been suggested to decrease the number of diffusion steps to solve this problem. For example, previous work^22,23^ suggested incorporating noise intensity into the model rather than time (as in^13,14^), which allows for greater flexibility in choosing the number and scheduling of diffusion steps and is effective for image super-resolution. Another intuitive approach to speeding up diffusion model sampling is to reduce the denoising step in the reverse process. However, previous research^14^ has shown that diffusion models often assume the denoising distribution learned during inverse synthesis can be approximated as a Gaussian distribution. This is problematic because the Gaussian assumption is only valid in the limit of many small denoising steps, which leads to slow synthesis in diffusion models. In this paper, we propose using a non-Gaussian multimodal distribution to model the denoising distribution when the reverse generation process uses larger step sizes (with fewer denoising steps).

## Experiment And Analysis

In this section, we will provide a detailed description of the experimental setup of the SRDDGAN model and demonstrate its effectiveness in the SISR task. Initially, we will briefly overview the dataset used, implementation details, and evaluation metrics. Subsequently, we will compare and analyze the experimental results of our model with those of other state-of-the-art models. Additionally, we conducted ablation experiments to explore the roles of various components in the proposed model. Finally, we will discuss the potential application value of this model in content fusion and the restoration of complex degraded images in real-world environments.

### Experimental Settings

**Datasets: **In the case of face super-resolution (8${\times }$), the same training data as SR3^22^ is utilized, consisting of 70,000 images from FFHQ^37^ and 28,000 images from CelebA-HQ^27^. The model is evaluated on 2000 images from CelebA-HQ. Following SR3, the HR images in the dataset are resized to 128${\times }$128 size. Subsequently, the HR images are downsampled using a bicubic kernel to generate an LR image of size 16${\times }$16.

For general task super-resolution (4${\times }$), the same training data as SRDiff is utilized, which includes 800 images from DIV2K^28^ and 2,650 images from Flickr2K^39^. The model is evaluated on 100 validation sets from DIV2K. During training and testing, each image in the dataset is cropped to 128${\times }$128 to obtain the HR image. The HR image is then downsampled using a bi-cubic kernel to generate an LR image of size 32${\times }$32. Additionally, for the general-purpose SISR task (2${\times }$), we utilized the CIFAR-10 dataset, which comprises 60,000 images across ten categories. During training and testing, each image in the dataset (32${\times }$32) was downsampled using bicubic interpolation to (16${\times }$16) resolution.

Finally, to address the diverse degradation modes in authentic images and enhance the model’s robustness, we applied a complex degradation algorithm mentioned in the second section to the low-resolution (LR) images. This algorithm involves random permutations of blurring, downsampling, and noise.

**Implementation details: **The experimental configuration remains identical for both face SR and general SR tasks, while the settings for other components are detailed in Table 1. The entire model training process was carried out using 4 TITAN V 12GB and 4 3090 24GB, and the model evaluation was done using GeForce GTX 1070 8GB. Table 1 in the paper shows the model parameter settings used for training and testing the CelebA-HQ, FFHQ, DIV2K, and Flickr2K datasets. These settings are consistent throughout the entire table. The same settings were also used for all the variants of the SRDDGAN in the ablation experiments.

**Table 1:** Training parameter settings of the model.

Training Config	FFHQ/CelebA-HQ	DIV2K/Flickr2K	CIFAR10
High-Resolution Size	128 $\times$ 128	128 $\times$ 128	32 $\times$ 32
Low-Resolution Size	16 $\times$ 16	32 $\times$ 32	16 $\times$ 16
Inner Channel	64	64	128
Channel Multiplier	(1, 1, 2, 2, 4, 4)	(1, 1, 2, 2, 4, 4)	(1, 2, 2, 2)
Scale of attention block	16	16	16
Latent embedding dimension	256	256	256
Timestep	4	4	4
Learning rate for generator	$1.0\textrm{E}-04$	$1.0 \textrm{E}-04$	$1.5 \textrm{E}-04$
Learning rate for discriminator	$1.6 \textrm{E}-04$	$1.6 \textrm{E}-04$	$1.2 \textrm{E}-04$
Training iterations	240k	300k	280k
Exponential Moving Average (EMA)	0.999	0.999	0.999
Optimizer	Adamw^40^	Adamw	Adamw
Loss function weights	$\alpha , \beta , \eta =1,0.8,0.2$	$\alpha , \beta , \eta =1,0.8,0.2$	$\alpha , \beta , \eta =1,0.8,0.2$
Optimizer Momentum	$\beta _{1}, \beta _{2}=0.5,0.9$	$\beta _{1}, \beta _{2}=0.5,0.9$	$\beta _{1}, \beta _{2}=0.5,0.9$
Batch size	48	48	128

**Evaluation metrics: **We use classical metrics such as Peak Signal-to-Noise Ratio (PSNR) and Structural Similarity Index (SSIM)^41^ to assess the difference between the reconstructed SR and the original HR images. Additionally, we utilize Learned Perceptual Image Patch Similarity (LPIPS)^42^ and Low-Resolution Peak Signal-to-Noise Ratio (LR-PSNR)^17^ as evaluation metrics. LPIPS measures perceptual similarity by comparing image features rather than relying on pixel values. It is more consistent with human perception than traditional evaluation metrics based on pixel values such as PSNR and SSIM. LR-PSNR is a recent evaluation metric for super-resolution algorithms that calculates the PSNR between the downsampled SR image and the LR image, reflecting the consistency between the output of the super-resolution algorithm and the LR. Additionally, we have introduced the FID (Fréchet Inception Distance)^43^ and IS (Inception Score)^44^ metrics to assess the quality and diversity of the generated images. Finally, to evaluate the sampling speed, we measure the clock time required to process a single image on a GeForce GTX 1070 and the number of iterations needed to process a single image.

### Performance

In this section, we assess the effectiveness of SRDDGAN by comparing it with various cutting-edge super-resolution techniques on face super-resolution (8${\times }$) and general super-resolution (4${\times }$) tasks. The specifics of these baseline models’ configurations can be found in their original research papers. Furthermore, we gauge our model’s performance against these baseline models regarding sample quality, diversity, and sampling speed.

**Figure 6:**
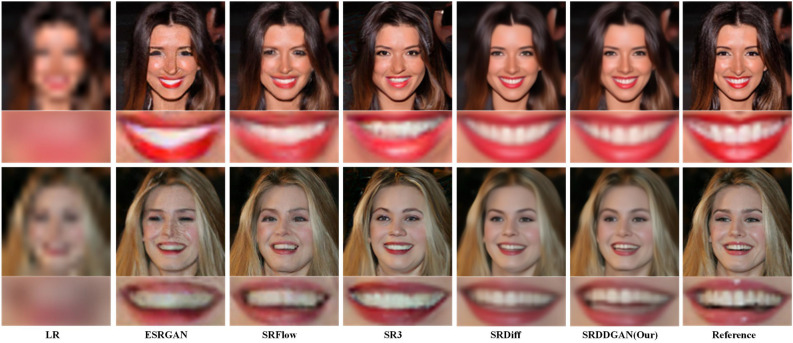
Face SR (8${\times }$) visual results. The SRDDGAN-generated details are more elaborate than those produced by SR3, SRFlow, and SRDiff. This approach circumvents the visual artifacts observed in ESRGAN, such as distortions in the woman’s teeth and eyes. Additionally, the SR produced by the model appears more realistic and diverse, maintaining consistency with the original image.

**Face SR:** Table 2 and Fig. 6 depict our evaluation of SRDDGAN on Face SR (8${\times }$) using the CelebA-HQ validation set. We benchmarked SRDDGAN against various state-of-the-art super-resolution models, namely PSNR-driven RRDB^38^ (which is a PSNR-oriented method trained using only L1 loss), GAN-based ESRGAN^38^, flow-based SRFlow^17^, and DDPM-based SR3^22^ and SRDiff^23^. The evaluation metrics show that in most cases, SRDDGAN outperforms the previous models, generating high-quality and diverse SR images that remain loyal to the LR consistency. Specifically :

1. According to Table 2, SRDDGAN demonstrates superior performance over other state-of-the-art super-resolution models in terms of perceived quality. LPIPS serves as a primary indicator in this comparison. SRDDGAN achieves nearly a 1${\times }$ improvement in the LPIPS score compared to RRDB, showcasing its superiority. Even compared to GAN-based methods, SRDDGAN achieves significantly better results on all reference indicators, including PSNR, which is traditionally considered a fidelity metric. This suggests that SRDDGAN maintains HR fidelity while also achieving better perceived quality. Compared with Flow-based and DDPM-based methods, we achieve some competitive performance on the reference metrics. Notably, SRDDGAN achieves the highest LR-PSNR score among all models, highlighting its consistency with the input LR image.
2. Figure 6 demonstrates that the SRDDGAN model outperforms ESRGAN in avoiding artifacts and preserving fine details, resulting in a precise and natural-looking image. Our model also produces superior visual results compared to SRDFlow in the tooth and eye regions. In addition, when compared to the DDPM-based method, SRDDGAN outperforms SR3 in the mouth area and generates more detailed results than SRDiff.
3. Our model (39.14M) has fewer parameters than SR3 (550M) and SRFlow (40M) while converging faster, taking only 240K iteration epochs in the same dataset to converge. In contrast, SRDiff convergence requires approximately 300K iteration epoch, and SR3 requires around 1000K iteration epoch, highlighting the high efficiency of our SRDDGAN model training.

**Table 2:** Results for 8${\times }$ SR of faces on CelebA-HQ.

Methods	PSNR$\uparrow$	SSIM$\uparrow$	LPIPS$\downarrow$	LR-PSNR$\uparrow$
Bicubic	23.38	0.65	0.484	34.66
RRDB^38^	26.89	0.78	0.220	48.01
ESRGAN^38^	23.24	0.66	0.115	39.91
SRFlow^17^	25.24	0.71	0.110	50.58
SR3^22^	23.04	0.66	0.098	47.00
SRDiff^23^	25.38	0.74	0.106	52.34
**SRDDGAN**	${\textbf {25.75}} _{\pm 0.0041}$	${\textbf {0.76}}_{\pm 0.0017}$	${\textbf {0.132}}_{\pm 0.0007}$	${\textbf {53.69}}_{\pm 0.0921}$

**Figure 7:**
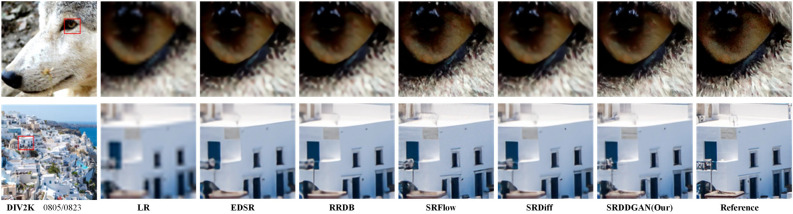
General SR (4${\times }$) visual results. SRDDGAN is superior to EDSR and RRDB in generating SR images that align with human perception instead of producing blurred hairs. Notably, only SRDDGAN successfully preserves the horizontal stripe on the brown wall in the second image, which corresponds with the reference image.

**General SR:** Table 3 and Fig. 7 display the outcomes of evaluating the generic SRDDGAN using the DIV2k validation set. The performance of SRDDGAN was compared with other models such as EDSR^45^, RRDB^38^, ESRGAN^38^, SRFlow^17^, and SRDiff^23^. For the 4${\times }$ setting, we used the officially released pre-trained models of these models for comparison. As a result, it was observed that SRDDGAN produced intricate details and exhibited excellent perceptual quality. Specifically:

1. As shown in Table 3, EDSR and RRDB models are trained exclusively using reconstruction losses, which results in subpar performance when evaluated based on the perceptual LPIPS metric. In contrast, our SRDDGAN model outperforms ESRGAN, which utilizes GANs in terms of PSNR, LPIPS, and LR-PSNR. Notably, SRDDGAN achieves the highest score in LR-PSNR among all other models;
2. In Fig. 7, it was noted that EDSR and RRDB produced unsatisfactory visualizations due to their inadequate generation of high-frequency details. Conversely, SRDDGAN surpassed SRDiff in perceptual quality by generating rich and detailed visualizations. Additionally, a close examination of the reference image revealed that SRDDGAN displayed superior perceptual details compared to SRFlow and SRDiff. In the first row, SRDDGAN produced intricate hair details in the top right corner of the eye and a sharp, brown horizontal line on the white wall in the second row.

**Table 3:** Results for 4${\times }$ SR of general images on DIV2K.

Methods	PSNR$\uparrow$	SSIM$\uparrow$	LPIPS$\downarrow$	LR-PSNR$\uparrow$
Bicubic	26.70	0.77	0.409	38.70
EDSR^45^	28.98	0.83	0.270	54.89
RRDB^38^	29.44	0.84	0.253	49.20
ESRGAN^38^	26.22	0.75	0.124	39.03
SRFlow^17^	27.09	0.76	0.120	49.96
SRDiff^23^	27.41	0.79	0.136	55.21
**SRDDGAN**	${\textbf {27.89}} _{\pm 0.0072}$	${\textbf {0.79}}_{\pm 0.0021}$	${\textbf {0.163}}_{\pm 0.0008}$	${\textbf {55.42}}_{\pm 0.0845}$

**High quality and diversity of sampling:** Assessing various models for the image super-resolution task on the CIFAR-10 dataset (2x upscaling), we evaluated their performance using the quantitative metrics in Table 4. Our SRDDGAN model exhibited outstanding performance in this task, delivering remarkable results. With an FID score of 3.92 on 50k CIFAR-10 images, SRDDGAN displayed exceptional image quality, competing competitively with top diffusion models and GANs. While LDM^46^ required 20000 diffusion steps for the same task, SRDDGAN only needed four steps, showcasing its rapid sampling speed. Furthermore, SRDDGAN achieved an IS score of 9.60, highlighting its outstanding image diversity, quality, and swift sampling performance. These findings underscore the excellent performance of SRDDGAN in image super-resolution tasks, offering robust support for high-quality, rapid sampling diverse image generation, demonstrating its potential and competitiveness in image processing.

Moreover, the results in Fig. 1 demonstrate that our model can generate diverse high-resolution (SR) images from a single low-resolution (LR) input image. These generated images exhibit natural variations in features such as hair tips, mouth shape, and eyebrow arches while remaining consistent with the input LR image.

**Table 4:** Quantitative comparison of SRDDGAN with state-of-the-art models on CIFAR-10 dataset ($\times 2$). FID and IS are computed on 50k samples.

Methods	Models	FID@50k$\downarrow$	IS$\uparrow$
VAE-based Methods	NVAE^47^	51.67	5.51
	D2C^48^	10.15	–
	DC-VAE^49^	17.90	8.20
Flow-based Methods	SRFlow	16.89	8.42
GAN-based Methods	AutoGAN^46^	5.29	8.55
	BigGAN^50^	14.73	9.22
	StyleGAN2^51^	8.3	9.21
	GLEAN^52^	13.78	8.34
DDPM-based Methods	NCSNV2^53^	10.87	8.40
	DiffFlow^54^	14.14	–
	LDM^55^	3.86	9.57
	**SRDDGAN**	${\textbf {3.92}} _{\pm 0.0608}$	${\textbf {9.60}} _{\pm 0.0404}$

**Figure 8:**
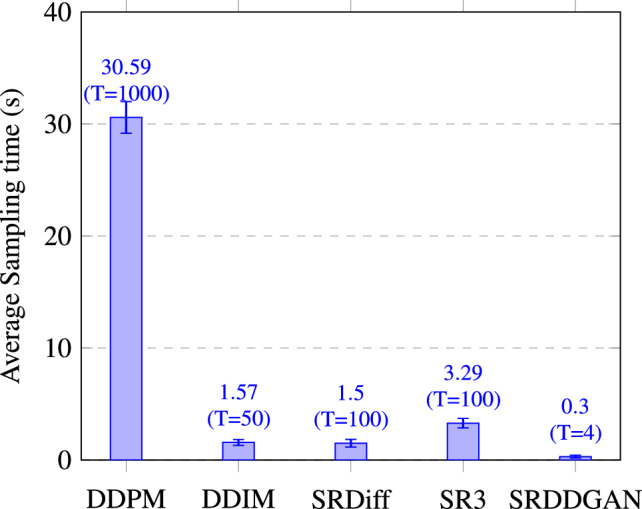
Comparison of sampling time and diffusion steps of different models on the CelebA-HQ dataset.

**Sampling speed and inference steps:** Figure 8 illustrates that the SRDDGAN model surpasses other diffusion-based image generation models, including DDIM^56^, an enhanced version of DDPM. The SRDDGAN model possesses two primary benefits: swifter sampling speed and superior image quality generation. Our model only requires 0.30 seconds to sample an image, whereas other diffusion-based image generation methods, such as SR3, demand 3.29 seconds per image sampling time. As a result, our model can produce more high-quality image samples in a shorter period. Additionally, our model shows an enhancement in PSNR evaluation metrics relative to SR3 and SRDiff (see Table 2). Notably, despite requiring just four sampling steps, our model achieves exceptional sample quality and speed, distinguishing us from other models.

### Ablation Study

We developed two models under low-resolution (LR) conditions and investigated their impact on Super-Resolution Deep Depth Generative Adversarial Networks (SRDDGAN). The first model (V$_{1}$ ) directly concatenates low-resolution images with noisy dimensions and feeds them into the model. The second model (V$_{2}$ ) extends this by incorporating a low-resolution encoder based on V$_{1}$ . Our research found that using a low-resolution encoder yields better performance metrics. Refer to the results in Table 5.

**Table 5:** The effectiveness of LR Encoder is verified on CelebA-HQ (8${\times }$).

Methods	PSNR$\uparrow$	SSIM$\uparrow$	LPIPS$\downarrow$	LR-PSNR$\uparrow$
**V**$_{1}$	$25.43 _{\pm 0.0073}$	$0.75 _{\pm 0.0051}$	$0.143 _{\pm 0.0025}$	$52.63 _{\pm 0.0878}$
**V**$_{2}$	${\textbf {25.50}} _{\pm 0.0067}$	${\textbf {0.75}} _{\pm 0.0060}$	${\textbf {0.140}} _{\pm 0.0028}$	${\textbf {52.81}} _{\pm 0.1188}$

As depicted in Table 6, the model in the 3 row demonstrates superior performance across all metrics, achieving a PSNR of 25.75, SSIM of 0.76, LPIPS of 0.132, and LR-PSNR of 53.69. The performance difference between the 2 and 3 rows is minor, but with the inclusion of content and style losses, the fourth row exhibits enhanced image quality and consistency. Introducing style and content losses significantly boosts the model’s performance, improving fidelity and perceptual similarity.

**Table 6:** Effectiveness of content and style loss for SRDDGAN on CelebA-HQ (8${\times }$).

Methods	PSNR$\uparrow$	SSIM$\uparrow$	LPIPS$\downarrow$	LR-PSNR$\uparrow$
SRDDGAN	$25.50 _{\pm 0.0067}$	$0.75 _{\pm 0.0060}$	$0.140 _{\pm 0.0028}$	$52.81 _{\pm 0.1188}$
SRDDGAN+$L_{content}$	$25.69 _{\pm 0.0054}$	$0.76_{\pm 0.0069}$	$0.134 _{\pm 0.0041}$	$53.12 _{\pm 0.0628}$
SRDDGAN+$L_{content}$+$L_{style}$	${\textbf {25.75}} _{\pm 0.0041}$	${\textbf {0.76}}_{\pm 0.0017}$	${\textbf {0.132}}_{\pm 0.0007}$	${\textbf {53.69}}_{\pm 0.0921}$

Table 7 presents the outcomes of a sequence of ablation experiments conducted to explore the impact of the size of the latent variable Z embedding dimension and the diffusion step size on the ablation of the diffusion model. We discovered from the data in rows 1, 4, 5, and 6 that the model generates higher quality and clearer images as the diffusion step size increases. Furthermore, rows 1, 2, and 3 illustrate that increasing the number of embedding dimensions of the latent variables enhances the quality of the super-resolved image and improves its agreement with the LR image. However, a larger diffusion step results in slower inference, and T=4 and Z=256 are set as the default settings to maintain consistency with LR images. The last row of Table 7 reveals that without any latent variable z, the model generates significantly poor sample quality, emphasizing the importance of multimodal denoising distributions.

**Table 7:** Ablations of SRDDGAN for faces SR on CelebA-HQ(8${\times }$).

Z	T	PSNR $\uparrow$	SSIM $\uparrow$	LPIPS $\downarrow$	LR-PSNR $\uparrow$
**256**	**4**	${\textbf {25.75}} _{\pm 0.0041}$	${\textbf {0.76}}_{\pm 0.0017}$	${\textbf {0.132}}_{\pm 0.0007}$	${\textbf {53.69}}_{\pm 0.0921}$
64	4	$25.67 _{\pm 0.0017}$	$0.76_{\pm 0.0013}$	$0.134_{\pm 0.0011}$	$51.43_{\pm 0.0244}$
128	4	$25.70 _{\pm 0.0039}$	$0.76_{\pm 0.0007}$	$0.128_{\pm 0.0004}$	$52.39_{\pm 0.0574}$
256	1	$22.45 _{\pm 0.0030}$	$0.67_{\pm 0.0023}$	$0.127_{\pm 0.0016}$	$44.76_{\pm 0.0577}$
256	2	$23.73 _{\pm 0.0019}$	$0.68_{\pm 0.0024}$	$0.133_{\pm 0.0002}$	$47.68_{\pm 0.1163}$
256	8	$25.95 _{\pm 0.0016}$	$0.77_{\pm 0.0020}$	$0.176_{\pm 0.0005}$	$49.78_{\pm 0.6871}$
0	4	$24.96 _{\pm 0.0029}$	$0.75_{\pm 0.0020}$	$0.112_{\pm 0.0008}$	$50.26_{\pm 0.1641}$

### Extensions

To comprehensively evaluate the model performance of SRDDGAN, we apply it in the domain of content fusion and real-world degraded pictures in this subsection.

**Figure 9:**
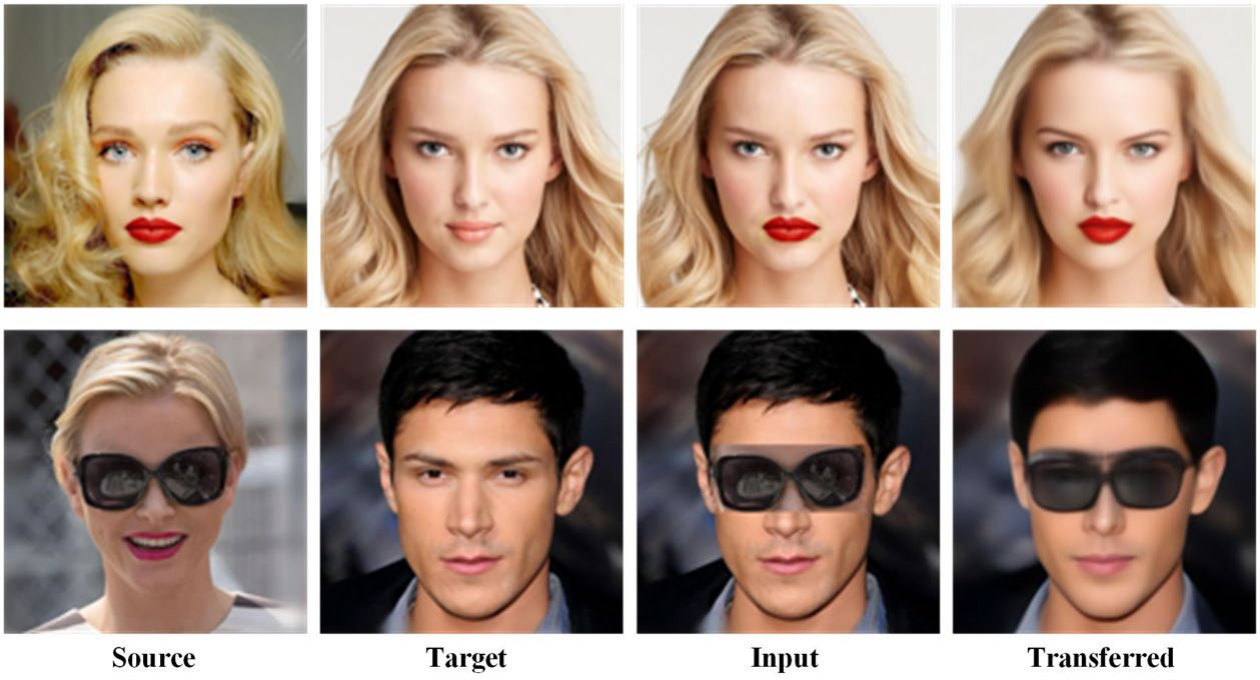
SRDDGAN model integrates and coordinates the content from the source image with the target image.

**Content fusion:**We aim to utilize other images to modify SR images. Let x represent an LR image, and y represent an HR image. If we are manipulating a super-resolved image, then ${ y}_{0} =G\left( x,y_{t} ,t,z\right)$ is an SR sample of x. However, we can also control an existing HR image y by setting $x=d\downarrow (x)$ to the down-scaled version of y. Subsequently, we can modify the SR image by directly incorporating additional image content in the image space. The forthcoming example illustrates merging one person’s eyes with the rest of another person’s face. The specific process of content fusion involves the following steps: Initially, we replace the source region image of the mouth (source) with the corresponding mouth region of the source image of the face (target) to generate a synthetic content image (Input). Subsequently, we obtained the LR image through bicubic downsampling and generated the corresponding SR image through model iteration. Lastly, we replace the mouth region on the source image with the corresponding mouth region on the target source image while preserving the unprocessed facial area. Figure 9 in the example showcases the transfer of facial features and eyes. The latent variable Z in our approach enhances the diversity of the generated SR image. For instance, in comparison to the source image, the mouth area of the sampled SR image is more varied and natural.

**Figure 10:**
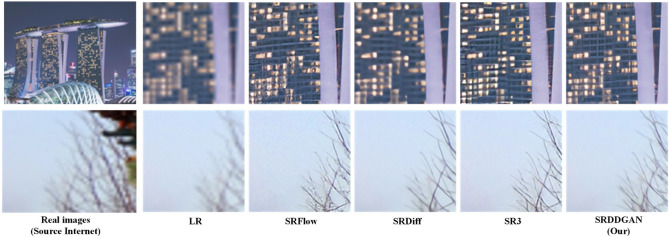
Real-world performance of SRDDGAN versus other state-of-the-art models.

**Experimental comparison on real-world datasets:**To comprehensively evaluate the capability of SRDDGAN in processing complex degraded images from the real world, we collected low-resolution (LR) images from actual environments. As shown in Fig. 10, the quality of high-resolution (HR) images reconstructed by SRDDGAN is significantly superior to those reconstructed by SRFlow, SRDiff, and SR3. Specifically, SRDDGAN in Fig. 10 is significantly better than the other models in detail and texture. For instance, the lines on the wall in the first row should be straight, and the branching of the tree limbs should be clear rather than blurred. In contrast, SRDDGAN reconstructs clear images and restores complete details and textures. Experiments on real datasets demonstrate that SRDDGAN has excellent generalizability and is suitable for single-image super-resolution (SISR) tasks in real-world scenarios.

## Conclusion

This paper introduces SRDDGAN, the first diffusion-based Single Image Super-Resolution (SISR) method model that relies on a small number of sampling steps. The study posits that in diffusion-based SISR tasks, the slow sampling speed is primarily due to the Gaussian assumption used in denoising distributions, which employs very few denoising steps. To address this issue, SRDDGAN is proposed. This method utilizes complex multimodal distributions to model each denoising step, allowing for more giant denoising strides. To alleviate the ill-posedness of super-resolution, latent variable Z is introduced to diversify the predictions of SR. Furthermore, to exploit the adequate information on Low-Resolution (LR) efficiently, a custom LR encoder module is employed to constrain the solution space of HR using a simple conditional generation approach. Finally, style and content loss functions are combined to recover some high-frequency details.

Many experiments show that SRDDGAN can generate a wide range of high-quality, realistic SR images. Moreover, these models demonstrate cost-effectiveness in testing, making them more practical for real-world applications. Despite exhibiting advantages in experiments, SRDDGAN still has limitations. For instance, it tends to produce blurry results, especially in the detailed texture of features such as hair, as seen in Figs. 6 and 9.

In the future, we plan to enhance the treatment of fine texture details without altering the existing diffusion steps. Initially, the image super-resolution reconstruction process will be divided into two stages. The initial stage prioritizes upsampling, utilizing networks like RRDB to enlarge low-resolution images and obtain the initial stage’s super-resolved images. In the second stage, we aim to restore residual maps of texture details, introducing residual learning and enhancing the fusion of super-resolution networks (texture transfer networks) with existing diffusion models to grasp and recover texture details. Finally, by combining the super-resolved images generated in the first stage with the residual maps from the second stage, we aim to develop the ultimate super-resolved photos to address the limitations observed in current super-resolution experiments. Furthermore, we aim to broaden the research to encompass a broader range of image transformation tasks, such as medical imaging, image coloring, and JPEG restoration.

### Supplementary Information

Supplementary Information.

## Data Availability

The dataset and code used and analyzed during the current study are available from the corresponding author upon reasonable request. The CelebA-HQ dataset is accessible on GitHub at https://github.com/tkarras/progressive_growing_of_gans. The FFHQ dataset can be found at https://github.com/NVlabs/ffhq-dataset. CIFAR-10 data is available via https://www.cs.toronto.edu/~kriz/cifar.html. The Flickr2K dataset can be obtained from http://cv.snu.ac.kr/research/EDSR/Flickr2K.tar. The Div2K dataset is accessible at https://data.vision.ee.ethz.ch/cvl/DIV2K/.
