# Supplementary material for: Single image super-resolution with denoising diffusion GANS
Source: Sci Rep. 2024 Feb 21;14:4272. doi: 10.1038/s41598-024-52370-3 (PMC11222509; doi:10.1038/s41598-024-52370-3)
Supplement: Supplementary file 1 — Supplementary Information. [file 41598_2024_52370_MOESM1_ESM.pdf]

Appendix

In this section, we provide further details about the architecture of the models used for super-resolution.

Details of Model Architecture for Super-Resolution

Table 1and Table 2 outline the key architectural features of the generator and discriminator used in our super-resolution task(where Table 2 displays the number of channels present in each residual block, as indicated by the number on the right-hand side.). We employ the same architecture for both face and general super-resolution tasks. Our method for conditioning the diffusion model on low-resolution images is illustrated in Fig. 1. First, we interpolate the low-resolution (16×16 or 32×32) image to the target high-resolution (128*128) and then concatenate it with the input noisy high-resolution image.

**Training Config**	**FFHQ/CelebA-HQ**	**DIV2K/Flickr2K**	**CIFAR10**
High-Resolution Size	128 × 128	128 × 128	32 × 32
Low-Resolution Size	16 × 16	32 × 32	16 × 16
Inner Channel	64	64	128
Channel Multiplier	(1, 1, 2, 2, 4, 4)	(1, 1, 2, 2, 4, 4)	(1, 2, 2, 2)
Scale of attention block	16	16	16
Latent embedding dimension	256	256	256
Timestep	4	4	4
Learning rate for generator	1.0E − 04	1.0E − 04	1.5E − 04
Learning rate for discriminator	1.6E − 04	1.6E − 04	1.2E − 04
Training iterations	240k	300k	280k
Exponential Moving Average (EMA)	0.999	0.999	0.999
Optimizer	Adamw[?]	Adamw	Adamw
Loss function weights	$\alpha, \beta, \eta = 1, 0.8, 0.2$	$\alpha, \beta, \eta = 1, 0.8, 0.2$	$\alpha, \beta, \eta = 1, 0.8, 0.2$
Optimizer Momentum	$\beta_1, \beta_2 = 0.5, 0.9$	$\beta_1, \beta_2 = 0.5, 0.9$	$\beta_1, \beta_2 = 0.5, 0.9$
Batch size	48	48	128

Table 1: Training parameter settings of the model

**CelebA-HQ / DIV2K**
1 × 1 Conv2d, 128
ResBlock down, 256
5× ResBlock down, 512
minibatch std layer
Global Sum Pooling
FC layer → scalar

Table 2: Discriminator

The setting of β_t in the diffusion process

In this section, we will discuss the setting of parameter β_t in the diffusion process. Given that we have set T to a relatively small value ($T <= 8$), we can utilize Variance Preserving Stochastic Differential Equation (VPSDE) to determine the magnitude of β_t for each step. Utilizing the continuous-time diffusion model formula to calculate β_t is advantageous since it maintains the variance's schedule and remains unaffected by the number of diffusion steps. The variance function of VPSDE is expressed as:

$$\sigma^2(t') = 1 - e^{-\beta_{\min} t' - 0.5(\beta_{\max} - \beta_{\min}) t'^2} \tag{A.1}$$

where $t' := t/T$ represents the normalized temporal variable that normalizes t from [1,2,..., T] to [0,1]. To match DDPM's configuration, we set $\beta_{\min}$ to 0.1 and $\beta_{\max}$ to 20. According to Eq. 11, we can perform a $q(y_t \mid y_0)$ sampling process within the forward diffusion process. It is important to note that the variance setting within $q(y_t \mid y_0)$ is equivalent to VPSED's variance, implying that we may calculate β_t via $\sigma^2(t') = 1 - \bar{\alpha}_t$.

$$\beta_t = 1 - \alpha_t = 1 - \frac{\bar{\alpha}_t}{\bar{\alpha}_{t-1}} = 1 - \frac{1 - \sigma^2\left(\frac{t}{T}\right)}{1 - \sigma^2\left(\frac{t-1}{T}\right)} = 1 - e^{-\beta_{\min}\left(\frac{1}{T}\right) - 0.5(\beta_{\max} - \beta_{\min})\frac{2t-1}{T^2}} \tag{A.2}$$

Based on the above formula, we can calculate the β_t value at each step.

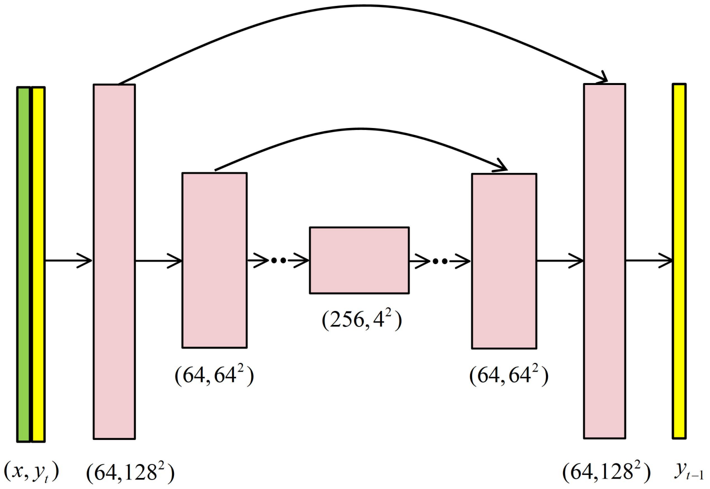

Figure 1: The generator architecture that utilizes U-Net and skips connections can be described as follows. The low-resolution input image, denoted by x, is first interpolated to match the target high-resolution and then combined with the noisy high-resolution image y_t. In the case of a super-resolution task from 16×16 to 128×128, the activation dimensions are displayed.
